# miRNA–mRNA Integrated Analysis Reveals Roles for miRNAs in a Typical Halophyte, *Reaumuria soongorica*, during Seed Germination under Salt Stress

**DOI:** 10.3390/plants9030351

**Published:** 2020-03-10

**Authors:** Huilong Zhang, Xiaowei Liu, Xiuyan Yang, Haiwen Wu, Jianfeng Zhu, Huaxin Zhang

**Affiliations:** 1 Research Center of Saline and Alkali Land of State Forestry and Grassland Administration, Beijing 100091, China; hlzhang@caf.ac.cn (H.Z.); liuxiaoweicaf@163.com (X.L.); sueyxy@126.com (X.Y.); 13621143840@163.com (H.W.); 2 Tianjin Research Institute of Forestry of Chinese Academy of Forestry, Tianjin 300450, China

**Keywords:** *Reaumuria soongorica*, seed germination, microRNA, salt stress, high-throughput sequencing

## Abstract

MicroRNAs (miRNAs) are endogenous small RNAs that play a crucial role in plant growth, development, and environmental stress responses. *Reaumuria soongorica* is a typical halophyte that is widely distributed in saline–alkali desert regions. Under salt stress, *R. soongorica* can complete germination, a critical biological process in the life cycle of seed plants. To identify miRNAs and predict target mRNAs involved in seed germination during salt stress, nine small-RNA libraries were constructed and analyzed from *R. soongorica* seeds treated with various concentrations of NaCl. We also obtained transcriptome data under the same treatment conditions. Further analysis identified 88 conserved miRNAs representing 25 defined families and discovered 13 novel miRNAs from nine libraries. A co-expression analysis was performed on the same samples to identify putative miRNA–mRNA interactions that were responsive to salt stress. A comparative analysis of expression during germination under 273 (threshold) and 43 mM (optimal) NaCl treatments identified 13 differentially expressed miRNAs and 23 corresponding target mRNAs, while a comparison between 43 mM NaCl and non-salt-stress conditions uncovered one differentially expressed miRNA and one corresponding target mRNA. These results provide basic data for further study of molecular mechanisms involved in the germination of salt-stressed *R. soongorica* seeds, and also provide a reference for the improvement of salt tolerance during plant germination.

## 1. Introduction

Seed germination is a key event in the life cycle of seed plants. During germination, a seed in a stationary (dry seed) state is transformed into a metabolically active embryo that breaks through the cyst structure (the endosperm and testa) and forms a young seedling [1,2]. These early germination events can be affected by a wide range of biological and abiotic factors [1,2,3,4].

Salt stress is a major abiotic stress that limits seed germination [5]. *Reaumuria* (Tamaricaceae) plants are typical perennial, salt-secreting, halophyte shrubs. This genus contains 12 species, which are widely distributed in saline–alkali desert regions worldwide, especially in Asia, North Africa, and southern Europe. Four species are currently found in China, namely *R. soongorica*, *R. alternifolia*, *R. kaschgarica*, and *R. trigyna*. *Reaumuria soongorica* is a representative species of saline–alkali desert ecosystems throughout Central Asia, especially in Taklamakan, Gurbantungut, Kumtag, Bataan Khiran, Qaidam, southern Russia, Inner Mongolia, and the Tengger Desert [6,7]. Because of accelerating global climate change and human activity, salinization is increasing in these regions [6,8]. *Reaumuria soongorica* has endured the desertification and salinization of Asia that began at least 22 million years ago, as evidenced by palaeomagnetic measurements and the fossil record [9].

*Reaumuria soongorica* is widely distributed in the northwestern region of China, where it is a representative dominant species of steppe saline–alkali desert areas. The species is mainly distributed along salt lake coasts and in saline–alkali depressions [10]. *Reaumuria soongorica* occupies an important position in the ecological and economic zone community types of the Inner Mongolia Plateau [11] and possesses abundant foliar levels of proteins, fats, and micronutrients [12]. The species plays a crucial role in the stabilization of shifting sand and is thus extremely useful for reducing the effects of desertification and overgrazing—the major environmental problems in the Inner Mongolian region over the past 50 years [13,14,15]. The most important grazing lands in Inner Mongolia are usually those growing large quantities of *R. soongorica*, which can also serve as a salt source for livestock [12,16,17]. *Reaumuria soongorica* can start and complete the seed germination process in a high-salt environment [18,19]. Several research teams have completed studies on the physiology and biochemistry of *R. soongorica* under abiotic stress conditions [18,19,20,21,22,23,24,25], but none have focused on the roles of miRNAs during germination of salt-stressed *R. soongorica* seeds. High-throughput sequencing of small RNAs based on transcriptome data is an efficient method for studying miRNAs and their regulatory networks.

MicroRNAs are an important class of non-coding regulatory small (20–24 nt) RNAs that mediate gene expression by degrading target mRNAs or repressing mRNA translation [26]. Recent attention has been focused on elucidation of mechanisms used by miRNAs to regulate gene expression. Various studies have determined that miRNAs are widely involved in plant biological and metabolic processes, such as growth, development, and response to stress [27,28,29], including salinity stress [30]. Although thousands of miRNAs from different plant species have currently been identified by high-throughput sequencing technology and registered in miRBase22.1 (http://www.mirbase.org/), information about *R. soongorica* miRNAs is entirely absent from this database.

The objective of our study was to catalog *R. soongorica* miRNAs and identify putative miRNAs (and their corresponding target genes) responsive to salt stress during germination of *R. soongorica* seeds. We used high-throughput sequencing technology to analyze nine small-RNA sequencing libraries obtained from *R. soongorica* seeds exposed to no salt stress (CS), an optimal salt concentration for germination (LS), and a threshold salt concentration for germination (MS). We identified miRNAs and predicted their mRNA targets based on comparisons with corresponding expression profiles of the *R. soongorica* transcriptome. We were thus able to further reveal the regulatory mechanism of miRNAs and mRNAs under salt stress. Our generated data provide new insights into the regulation of germination of salt-stressed *R. soongorica* seeds and should spur the improvement of non-halophyte seeds for germination under salt stress. 

## 2. Results

### 2.1. Summary of the Small-RNA Library Dataset Obtained from Deep Sequencing of R. soongorica 

To explore the regulatory mechanism of miRNAs in the germination of salt-stressed seeds, we collected and sequenced total RNA from *R. soongorica* seeds grown under different salt concentrations: CS, without salt stress; LS, an optimal salt concentration for germination; and MS, a threshold salt concentration for germination. Using Illumina sequencing technology, approximately 44.90, 40.79, and 44.82 million raw reads were obtained from triplicate samples of CS, LS, and MS treatments, respectively (Table 1). Over 90% of raw reads remained after removal of adaptor sequences, low-quality tags, and reads longer than 30 nt or shorter than 18 nt. In total, we acquired nearly 125.86 million clean reads, more than 90% of which were subsequently mapped to the *R. soongorica* reference sequence (Table 2). 

**Figure 1:**
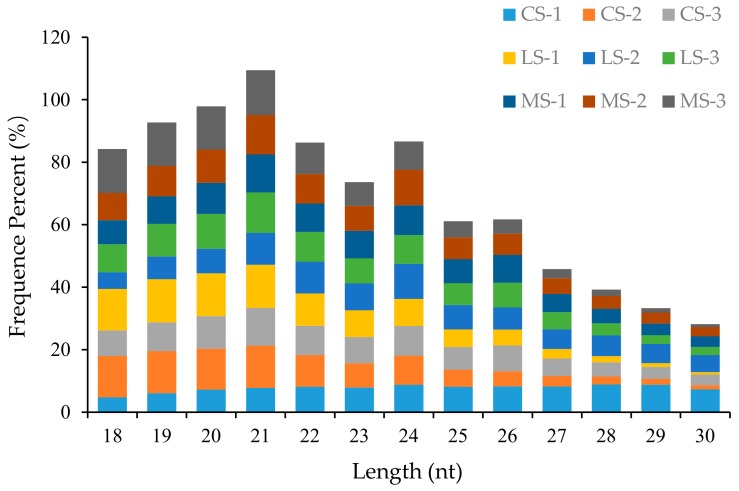
Small-RNA length distribution in *Reaumuria soongorica* seeds. CS, without salt stress; LS, optimum salt concentration for germination; MS, threshold salt concentration for germination.

Small-RNA sequences of 18 to 30 nt in length, the most abundant of which were 19–24 nt long, were selected for further study (Figure 1). These mapped small RNAs comprised known miRNAs, putative novel miRNAs, ribosomal RNA (rRNAs), transfer RNA (tRNAs), small nuclear RNA (snRNAs), small nucleolar RNA (snoRNAs), *trans*-acting small interfering RNAs (TAS), and unannotated fragments (Table 2). We found that 0.08%–1.14% of the mapped small RNAs were previously identified miRNAs, while 76.65%–85.02% were unannotated small RNAs. This high number of unannotated small RNAs suggests that additional miRNAs remain to be identified in *R. soongorica*. Prediction of novel miRNAs was based on unannotated small RNAs. The most abundant small-RNA sequence length was 21 nt (10.75%), followed by 20 nt (9.72%) and 19 nt (9.21%) (Figure 1). These lengths and relative populations are similar to typical small-RNA distributions in angiosperms.

### 2.2. Identification of Conserved miRNAs from Known Families

**Figure 2:**
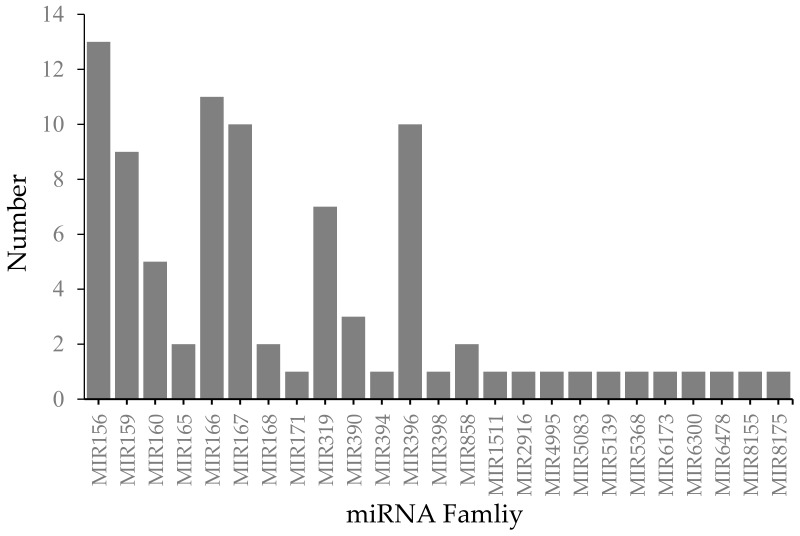
Numbers of conserved miRNAs per miRNA family in *Reaumuria soongorica* seeds. The x- and y-axes represent the different conserved miRNA families found in all nine libraries and the numbers of members in each family, respectively.

To identify conserved miRNAs in *R. soongorica* seeds, sequences of small RNAs were compared with known plant miRNA sequences in the miRBase database. Following a BLASTN search and further sequence analyses, we uncovered 88 miRNAs belonging to 25 previously identified miRNA families (Table S1). Approximately half of the families had more than one member. The largest family, MIR156, contained 13 members (Figure 2).

Obvious differences in abundance, as reflected by library read counts, were observed among these miRNA families (Table S1). To compare expression levels in this study, we calculated the average transcripts per million reads (TPM) of each miRNA in nine libraries [31,32,33]. After TPM standardization, rso-miR171a had the lowest expression level and a relatively low abundance (92.13 TPM), while the highest relative expression level was that of rso-miR159a (146,916.58 TPM), followed by rso-miR159c (76,138.88 TPM) and rso-miR159b (73,930.26 TPM).

### 2.3. Discovery of Novel miRNAs in R. soongorica Seeds

To identify novel miRNAs from the unannotated small-RNA tags, the miREvo and miRDeep2 programs were used to predict secondary structures and Dicer cleavage sites and to measure minimum free energies. In total, 13 putative novel miRNAs were predicted from the nine libraries (Table S2). All of these novel miRNAs were found in at least two independent libraries of a given treatment, and nine were found in all nine libraries. Novel mature miRNAs ranged in length from 18–24 nt, and approximately 60% were 21 nt long. Fewer novel miRNAs (13) were uncovered than conserved miRNAs (88). These putative novel miRNAs may represent new miRNA families or correspond to previously unknown members of known miRNA families.

### 2.4. Differential Expression of miRNAs during Germination of R. soongorica Seeds Exposed to Various Salt Treatments

To determine the relationship between miRNA expression and salt stress, *R. soongorica* seeds were treated with NaCl during germination. The normalized expressions of miRNA families in LS libraries were compared with those in CS and MS libraries using fold-change calculations. Any miRNA with an absolute fold change of greater than 1 as well as a *p*-value of less than 0.05 was considered to be differentially expressed under salt stress (Table S3) [34,35,36].

**Figure 3:**
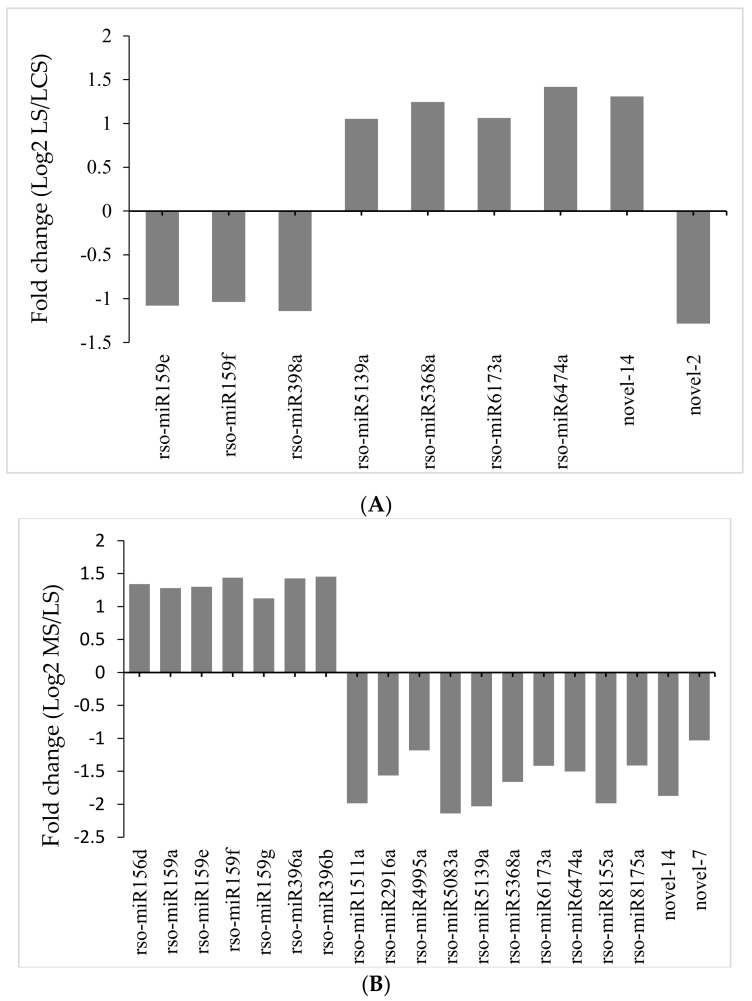
Comparison of relative expressions of differentially expressed miRNAs. (**A**) Relative expressions of miRNAs between LS (optimum salt concentration for germination) vs. CS (without salt stress) libraries. (**B**) Relative expressions of miRNAs between MS (threshold salt concentration for germination) and LS libraries.

Compared with their expressions in CS, four miRNAs were down-regulated and five were up-regulated in LS (Figure 3A), while 12 miRNAs were down-regulated and seven were up-regulated in MS compared with LS (Figure 3B). In addition, two novel miRNAs each were differentially expressed in LS vs. CS and MS vs. LS comparisons. These differences indicate that the expressions of these miRNAs dynamically respond to changing NaCl concentrations. Details of the differentially expressed novel and conserved miRNAs are summarized in Table S3. Taking into account the expression patterns of these miRNAs, we speculate that they play a crucial role in the germination of *R. soongorica* seeds under salt-stress conditions. Among them, several non-classical miRNAs and novel miRNAs, including rso-miR5139a, rso-miR6173a, and novel-14, have not been previously implicated in non-halophyte seed germination under salt stress [37,38], thus indicating that unique regulatory mechanisms may be used by halophytes.

### 2.5. miRNA and mRNA Correlation Analysis

**Figure 4:**
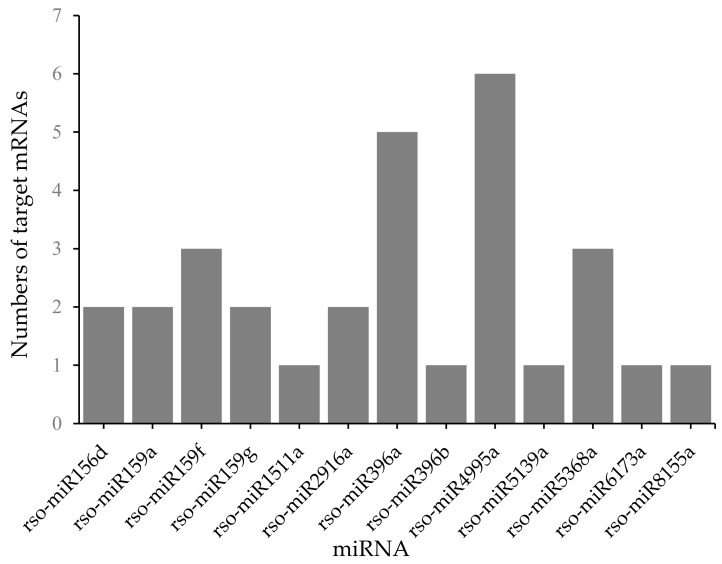
Numbers of mRNA targets of miRNAs based on an miRNA–mRNA correlation analysis of MS vs. LS. The y-axis indicates the number of target mRNAs in each miRNA, displayed on the x-axis, as revealed by the miRNA–mRNA correlation analysis. LS, optimum salt concentration for germination; MS, threshold salt concentration for germination.

To further explain the role of miRNAs in salt-stress response and confirm potential mRNA targets identified by sequencing, we performed an extensive correlation analysis of the expressions of miRNAs and their target mRNAs. Two miRNA–target–mRNA groups of clusters were identified from two comparisons. One group, identified by comparison of LS and CS expressions, consisted of a cluster containing a single miRNA (Table S4). The other group, which highlighted variations between MS and LS conditions, included 13 miRNAs and 23 target mRNAs. The largest cluster in this group was rso-miR4995a, which included six regulated mRNAs (Figure 4; Table S4). Overall, the fold-change expression levels of mRNAs were correlated with those of their paired miRNAs in both groups of clusters. In addition, connections including genes involved in transcriptional regulation can lead to significant secondary effects on the transcription of other genes. Details of the results of the miRNA–mRNA correlation analysis are found in Table S4.

## 3. Discussion

miRNAs are an important class of non-coding regulatory small RNAs involved in gene regulation in most eukaryotic cells. Many studies have indicated that miRNAs are widely involved in a variety of stress responses, including responses to drought, cold, salinity, oxidative stress, and viruses [39,40,41,42,43,44,45]. Consequently, tremendous efforts have been made to study miRNAs in various plant species. Traditional methods for discovering and identifying miRNAs, such as Sanger sequencing and bioinformatics prediction, are time-consuming and labor-intensive. In addition, this type of technology has difficulty detecting novel or low-abundance miRNAs. High-throughput sequencing technology, which is rapid and efficient, is ideal for the study of novel or low-abundance miRNAs and the analysis of small-RNA clusters at multiple stages of plant development under various natural environmental stress conditions [46,47]. 

Since the high-throughput sequencing’s first application in the model species *Arabidopsis thaliana* [48], it has played a huge role in the discovery of miRNAs in various plant species, including barley [31], rubber tree [33], peach [49], moso bamboo [50], peanut [51], *Nicotiana benthamiana* [52], and *Ulmus pumila* [53]. To date, 7474 hairpin sequences and 9168 mature miRNA sequences have been identified from 74 angiosperm species. Although these achievements have been incorporated into miRBase 22.1, sequence information for *R. soongorica* miRNAs is entirely absent from this database.

Seed germination begins with water absorption (swelling) that mobilizes stored materials, which in turn causes cells to elongate until the radicle breaks through the seed coat at the completion of germination. The mobilization of stored materials involves a series of metabolic reactions. In response to biotic and abiotic stress conditions, these reactions can be modulated by altering gene expression and by using different metabolic or signal transduction pathways. *Reaumuria soongorica*, a typical halophyte plant, may possess special regulatory mechanisms that enable its seeds to complete germination under salt conditions that prevent the germination of non-halophytes.

In the present study, we obtained a large number of small-RNA sequences from salt-stressed *R. soongorica* seeds. These sequences were analyzed using the miRBase database, enabling the identification of 88 conserved miRNAs belonging to 25 known families corresponding to the majority of miRNAs previously found in other plant species (Table 1). We detected 13, 11, 10, and 10 members of MIR156, MIR166, MIR167, and MIR396 families, respectively, in *R. soongorica*. In addition, we identified 13 novel miRNAs not previously detected in any known families.

In our earlier studies, we used transcriptome data to also identify genes differentially expressed in LS, CS, and MS conditions [18]. To better understand miRNA–mRNA regulatory relationships predicted by sequencing data, we compared our small-RNA expression data in the current study with those previously obtained for putative target mRNAs. An miRNA–mRNA correlation analysis was performed to visualize negatively and positively correlated interaction groups of clusters according to the method of Ma et al. (2011) [54]. 

The LS vs. CS comparison group consisted of one cluster, which included one differentially regulated miRNA (Table S4). The group comparing MS and LS samples included 13 miRNAs and 23 target mRNAs (Table S4). The auxin response factor gene was found to be a potential target of MIR396, an miRNA family involved in auxin signal transduction during many stages of plant growth development [45,55]. Compared with their expressions under LS conditions, rso-miR396a and rso-miR396b tended to be up-regulated in higher-salt MS conditions and were correlated with decreased expression of HB33 to inhibit seed germination. The gene encoding copper/zinc superoxide dismutase (the expected target of MIR398) plays an important role in plant response to abiotic stress [56]. The rso-miR398a was down-regulated by the increased salinity of the LS treatment compared with CS, but no significant difference was observed between MS and LS. This observation contrasts with the findings of studies on *A. thaliana*, *Ulmus pumila*, and *Caragana intermedia*, in which the expression of miR398 was reduced under salt stress [53,57,58]. This result suggests that a difference exists in halophyte and non-halophyte responses to low-concentration salt stress.

**Figure 5:**
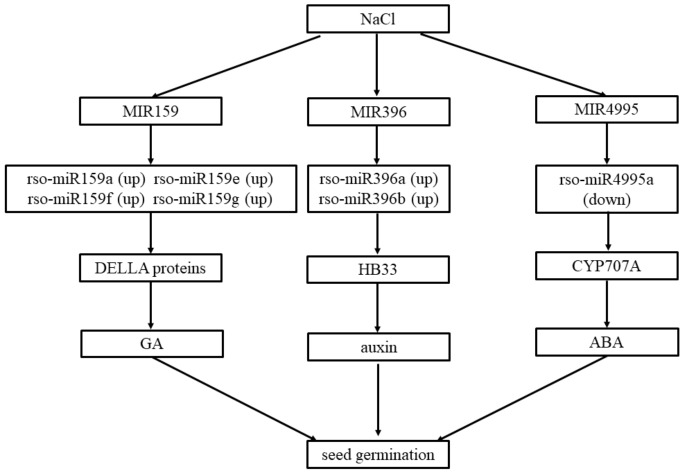
Hypothetical model of the miRNA regulatory network of seed germination response to salt stress in *Reaumuria soongorica*.

We also found that *CYP707A2*, a key gene encoding ABA 8′-hydroxylase in ABA (abscisic acid) catabolism [59], is the likely target of MIR4995. In Arabidopsis, *CYP707A* genes encode ABA 8′-hydroxylases and are involved in many physiological processes [60]. Previous experiments have demonstrated that ABA 8′-hydroxylase genes, such as *CYP707A1* and *CYP707A2* in Arabidopsis, play prominent roles in the regulation of endogenous ABA levels during seed dormancy and germination [59,60,61,62]. In contrast to results comparing LS with CS, rso-miR4995a exhibited a downward-regulated trend in MS relative to LS. MIR159 putatively targets mRNAs encoding GA (gibberellin)-MYB (v-myb avian myeloblastosis viral oncogene homolog) transcription factors that interact with GA-response elements and participate in short-day floral initiation and anther development [63,64]. Interestingly, the expression of miR159 is repressed in the absence of GA, possibly through the action of DELLA proteins (named for a conserved pentapeptide motif) that are in turn targeted for GA-induced proteolysis [63]. The MIR159 family exhibited an upward-regulated trend in MS relative to LS, which contrasts with its down-regulation in LS compared with CS. These results may reflect a unique regulatory mechanism used by halophyte seeds during germination under salt stress. On the basis of our results and previous research, we hypothesize that *R. soongorica* quickly activates miRNA regulatory mechanisms under salt stress and further regulates GA, auxin, and ABA pathways, thereby regulating the germination ability of its seeds under salt stress (Figure 5). These results indicate that *R. soongorica* may inhibit seed germination by reducing auxin and gibberellin levels and increasing ABA concentrations under high salinity. This regulatory mechanism ensures that *R. soongorica* halts germination under high salt stress but can survive periods of high salinity (i.e., salt concentrations exceeding the level that *R. soongorica* can tolerate during the germination period) and germinate under low-salt conditions (i.e., within the salt-stress concentration range that *R. soongorica* can tolerate during the germination period) to improve survival.

Our study is the first to identify miRNAs and target genes specifically implicated in the germination of salt-stressed *R. soongorica* seeds. Our results provide an overview of potential pathways and mechanisms involved in this process. Nevertheless, more experiments are required to confirm the targets of differential miRNA expression and clarify the regulatory gene groups of clusters underlying subsequent regulation of germination of salt-stressed *R. soongorica* seeds.

## 4. Materials and Methods 

### 4.1. Plant Material and Salt-Stress Treatment

*Reaumuria soongorica* seeds (1000-seed weight = 1.137 ± 0.121 g) were collected from saline desert areas of the semi-desert steppe of the western Inner Mongolian Plateau, China. *Reaumuria soongorica* is a diploid with a chromosome number of 22. Karyotypic analysis has determined that chromosome sizes range from 3.38 to 5.51 μm, and the chromosomal formula is 2*n* = 2*x* = 22 = 4m + 14sm + 4st. Fluorescent in situ hybridization detected four pairs of 45S rDNA signals at the ends of *R. soongorica* chromosomes. In addition, a flow cytometry analysis predicted a genome size of approximately 778 Mb [65]. The natural salt composition of the soil in the collection area is mainly chloride–sulfate, of which NaCl is an important component and the most harmful to plant growth. The total soil salt content is generally 0.3%–0.6%; during the spring seed-germination period, however, the rate of soil-surface salt accumulation is as high as 100%, and the total salt content can reach 0.6%–1.2% [66,67,68]. 

We carried out germination experiments on *R. soongorica* seeds in the presence of different NaCl concentrations, observed *R. soongorica* growth and physiological indicators during the germination period under salt stress, and calculated the salt tolerance threshold and optimal salt-stress concentration [18,19]. We obtained a salt tolerance threshold of 273 mM (corresponding to a salt content of 1.58%). This concentration of NaCl, the main type of salt in the habitat of *R. soongorica*, is slightly higher than the average maximum total salt concentration recorded in spring; because this concentration should therefore generate the optimal stress effect while simulating natural conditions, it was applied as a treatment in this study (denoted as MS). By observing the growth performance of seeds under salt stress, we also determined that the optimum NaCl treatment concentration was 43 mM; denoted as LS, this treatment can promote the germination and growth of *R. soongorica* seeds [18,19].

The *R. soongorica* seeds were placed on a double layer of filter paper in a culture dish and germinated under MS, LS, or distilled water (defined as CS) conditions at 25 °C and 55% humidity in the dark. The treatments were repeated three times. When a hypocotyl had broken through the seed coat but did yet not exceed 2 mm in length, it was harvested, immediately frozen in liquid nitrogen, and stored at −80 °C until use in small-RNA library construction.

### 4.2. Small-RNA Library Construction and Sequencing

Samples were ground to a powder under liquid nitrogen, and aliquots of approximately 100 mg were placed in sterile 2 mL Eppendorf tubes. After addition of a suitable amount of Trizol reagent (Invitrogen, Carlsbad, CA, USA), the contents of each tube were homogenized for three minutes using a vortex shaker, and total RNA was extracted according to the Trizol kit instructions. RNA integrity was checked by 1% agarose gel electrophoresis and on a bioanalyzer (Agilent 2100, Santa Clara, CA, USA), with only those samples with an RNA integrity value of ≥7.5 used for subsequent analysis. Nine small-RNA libraries (CS-1, CS-2, CS-3, LS-1, LS-2, LS-3, MS-1, MS-2, and MS-3) were constructed from the purified RNA using an NEB Multiplex Small RNA Library Preparation kit (NEB, Ipswich, MA, USA). Index codes were added to attribute sequences to each sample. Briefly, specific adapters were added to the 3′ and 5′ ends of miRNA, small interfering RNA, and piwi-interacting RNA, and first-strand cDNA was then synthesized using M-MuLV reverse transcriptase (RNase H-) as a catalyst. PCR amplification was performed with specific primers and LongAmp *Taq* 2x Master Mix (Illumina, San Diego, CA, USA). PCR products were separated by electrophoresis on an 8% polyacrylamide gel at 100 V for 80 min. Recovered DNA fragments of the expected length (140–160 bp) were dissolved in 8 µL of elution buffer. Library quality was assessed on an Agilent Bioanalyzer 2100 system using high-sensitivity DNA chips. Clustering of the index-coded samples was performed on a cBotCluster Generation System using a TruSeq SR Cluster Kit v3-cBot-HS (Illumina) following the manufacturer’s instructions. After completing the above pretreatments, the purified library was sequenced on an Illumina Hiseq 2500 platform by Novogene (Beijing, China). The small-RNA sequencing data collected in this study have been deposited in the NCBI Sequence Read Archive (SRA) database under accession number PRJNA578509.

### 4.3. Identification of Conserved and Novel miRNAs

After sequencing, clean reads were obtained from the raw data by removing adapter-contaminated or low-quality reads, poly-N-containing reads, and reads shorter than 18 nt. All remaining sequences were considered to be of high quality and were further analyzed. Because no *R. soongorica* genome has been published and nucleic acid sequences are lacking in known public databases, a transcriptome (poly[A]-enriched mRNA) of *R. soongorica* seeds germinated under the same salt treatment conditions was sequenced as a reference (SRA database: PRJNA510727) [21]. Because pre-miRNA sequences are very short and lack poly(A) tails, they cannot be captured by transcriptome sequencing; consequently, the miRNA sequences we obtained in this study were likely pri-miRNAs [69]. Using bioinformatics methods, pre-miRNA sequences can be identified on the basis of their characteristic hairpin structures [70,71]. Only those sequences that could be mapped to the transcriptome sequence using Bowtie (http://banana-geno-me.cirad.fr/content/download-dh-pahang) [72] were analyzed further. Sequences matching non-coding RNAs (rRNAs, scRNAs, snoRNAs, snRNAs, and tRNAs) in the Rfam 10.1 database were removed, and the remaining sequences were compared against known miRNAs in miRBase 22.1 (http://www.mirbase.org/). To predict novel miRNAs, unannotated sRNA sequences were analyzed for hairpin structures, Dicer cleavage sites, and minimum free energy with an integrated combination of miREvo [73] and mirdeep2 [71] software. The criteria used for identifying novel miRNAs were based on Meyers et al. (2008) [74]. Those miRNAs showing extremely low expression levels (≤2 reads in any two treatment repeats [34]) in the nine libraries were removed and not used in further analyses.

### 4.4. Relative Expression between miRNA Libraries 

To analyze the differential accumulation of miRNAs in the nine libraries, miRNA expression levels, including those of conserved and novel individual miRNAs, were calculated as TPM (per million transcripts) [43]. The *p*-values for comparisons of expression profiles between different miRNA libraries were obtained using the DESeq2 R package [75,76,77]. The differentially expressed miRNAs were screened using the following thresholds: Absolute fold change greater than 1 as well as a *p*-value less than 0.05, generally the highest acceptable significant *p*-value; miRNAs matching these criteria were considered to be up-regulated or down-regulated, respectively, in response to salt stress [75,76,77].

### 4.5. Prediction of miRNA Target Genes 

Sequence complementarity between miRNAs and potential target mRNAs was assessed using computational analysis [78]. Target prediction was carried out using the psRobot tool based on psRobot_tar [79] and through alignment with *R. soongorica* transcriptomes collected under the same experimental conditions (SRA database: PRJNA510727) [18]. The extracted sequences were further analyzed using the NCBI database to obtain possible protein-coding genes in *R. soongorica*. 

### 4.6. miRNA and mRNA Correlation Analysis 

To better understand predicted miRNA–mRNA regulatory relationships, we constructed groups of clusters containing miRNA–mRNA pairs negatively and positively correlated in the small-RNA and transcriptome expression datasets. First, we screened the miRNA data to obtain miRNAs that could match mRNA and identified mRNAs that included targeted miRNAs based on the *R. soongorica* transcriptome data. Finally, we performed a correlation analysis of the two databases obtained above to identify the miRNA–mRNA regulatory relationship [54,80].

## 5. Conclusions

In this study, we identified 88 conserved miRNAs representing 25 defined families and discovered 13 novel miRNAs from *R. soongorica* using small-RNA high-throughput sequencing technology. Co-expression analysis of putative miRNA–mRNA interactions using samples acquired at varying salt treatments allowed us to obtain two groups of clusters that connected one miRNA with one mRNA target in LS vs. CS and 13 miRNAs with 23 target mRNAs in MS vs. LS. Our results provide a foundation for further research on the molecular mechanisms of seed germination of *R. soongorica* during salt stress and can also serve as a basis for improving plant salt tolerance during germination.

## Figures and Tables

**Table 1:** Results of sRNA sequencing of *Reaumuria soongorica* seed libraries.

Category	Raw Reads	N% > 10%	Low Quality Reads	5′ Adapter Contamine	3′ Adapter Null	Poly (A/T/G/C)	Unique Reads	Clean Reads
CS-1	13,869,156 (100.00%)	564 (0.00%)	19,225 (0.14%)	33,490 (0.24%)	224,816 (1.62%)	12,005 (0.09%)	1,315,057	13,579,056 (97.91%)
CS-2	16,819,324 (100.00%)	639 (0.00%)	21,867 (0.13%)	15,406 (0.09%)	964,063 (5.73%)	7615 (0.05%)	1,376,042	15,809,734 (94.00%)
CS-3	14,212,954 (100.00%)	543 (0.00%)	16,330 (0.11%)	14,962 (0.11%)	398,880 (2.81%)	7654 (0.05%)	1,156,054	13,774,585 (96.92%)
LS-1	14,552,453 (100.00%)	527 (0.00%)	18,159 (0.12%)	13,581 (0.09%)	702,302 (4.83%)	4291 (0.03%)	993,154	13,813,593 (94.92%)
LS-2	12,328,793 (100.00%)	451 (0.00%)	19,864 (0.16%)	13,884 (0.11%)	179,427 (1.46%)	7466 (0.06%)	1,442,730	12,107,701 (98.21%)
LS-3	13,908,660 (100.00%)	504 (0.00%)	15,636 (0.11%)	11,487 (0.08%)	290,553 (2.09%)	4276 (0.03%)	818,193	13,586,204 (97.68%)
MS-1	12,887,720 (100.00%)	535 (0.00%)	19,878 (0.15%)	8991 (0.07%)	270,670 (2.10%)	3690 (0.03%)	1,014,692	12,583,956 (97.64%)
MS-2	17,338,149 (100.00%)	680 (0.00%)	23,377 (0.13%)	17,416 (0.10%)	490,306 (2.83%)	12,336 (0.07%)	2,026,130	16,794,034 (96.86%)
MS-3	14,594,879 (100.00%)	532 (0.00%)	15,790 (0.11%)	13,876 (0.10%)	743,198 (5.09%)	5511 (0.04%)	1,145,458	13,815,972 (94.66%)

CS, without salt stress; LS, optimal salt concentration for germination; MS, threshold salt concentration for germination.

**Table 2:** Numbers of reads classified according to the types of identified small RNAs.

Read Types	Total	Sequences Mapped to Genome	Known miRNA	Novel miRNA	Ribosomal RNA	Transfer RNA	Small Nuclear RNA	Small Nucleolar RNA	Trans-Acting Small Interfering RNAs	Unannotated
CS-1	9,127,130	8,474,172 (92.85%)	12,039 (0.14%)	1244 (0.01%)	1,245,902 (14.70%)	10 (0.00%)	2883 (0.03%)	6680 (0.08%)	641 (0.01%)	7,204,773 (85.02%)
CS-2	8,007,781	7,381,145 (92.17%)	61,754 (0.84%)	4490 (0.06%)	1,644,956 (22.29%)	15 (0.00%)	3419 (0.05%)	5609 (0.08%)	3583 (0.05%)	5,657,319 (76.65%)
CS-3	9,375,007	8,975,019 (95.73%)	20,397 (0.23%)	1423 (0.02%)	1,497,323 (16.68%)	5 (0.00%)	2433 (0.03%)	3034 (0.03%)	1038 (0.01%)	7,449,366 (83.00%)
LS-1	7,270,911	6,886,984 (94.72%)	16,112 (0.23%)	1515 (0.02%)	1,366,452 (19.84%)	9 (0.00%)	3193 (0.05%)	5135 (0.07%)	1646 (0.02%)	5,492,922 (79.76%)
LS-2	8,520,845	7,737,807 (90.81%)	32,024 (0.41%)	3086 (0.04%)	1,356,215 (17.53%)	9 (0.00%)	2832 (0.04%)	7026 (0.09%)	2159 (0.03%)	6,334,456 (81.86%)
LS-3	9,018,168	8,779,523 (97.35%)	7119 (0.08%)	604 (0.01%)	1,482,050 (16.88%)	6 (0.00%)	2286 (0.03%)	3677 (0.04%)	419 (0.00%)	7,283,362 (82.96%)
MS-1	8,981,640	8,581,120 (95.54%)	5456 (0.06%)	138 (0.00%)	1,452,970 (16.93%)	7 (0.00%)	2749 (0.03%)	1702 (0.02%)	121 (0.00%)	7,117,977 (82.95%)
MS-2	10,925,787	9,849,558 (90.15%)	112,529 (1.14%)	4902 (0.05%)	1,888,880 (19.18%)	10 (0.00%)	4224 (0.04%)	5038 (0.05%)	6472 (0.07%)	7,827,503 (79.47%)
MS-3	6,096,367	5,522,031 (90.58%)	51,689 (0.94%)	2498 (0.05%)	927,311 (16.79%)	9 (0.00%)	1767 (0.03%)	4458 (0.08%)	3196 (0.06%)	4,531,103 (82.06%)

CS, without salt stress; LS, optimal salt concentration for germination; MS, threshold salt concentration for germination.

## Supplementary Materials

The following information is available online at https://www.mdpi.com/2223-7747/9/3/351/s1. Table S1: Conserved miRNAs identified in *Reaumuria soongorica*; Table S2: Novel miRNAs identified in *R. soongorica*; Table S3: All (known and novel) differentially expressed miRNAs in LS vs. CS and MS vs. LS; Table S4: The results of miRNA–mRNA comparative analyses of LS vs. CS and MS vs. LS.
